# Parameter Estimation Using Divide-and-Conquer Methods for Differential Equation Models

**DOI:** 10.4172/2155-6180.1000305

**Published:** 2016-05-30

**Authors:** Seongho Kim

**Affiliations:** Biostatistics Core, Karmanos Cancer Institute, Department of Oncology, School of Medicine, Wayne State University, USA

In systems biology, a key topic is the elucidation of the dynamic behavior of biological processes that are made up of complex biochemical networks. Statistical modeling is an important to capture the dynamics of biochemical networks such as metabolic networks, signal transduction pathways, and gene regulatory networks. These biochemical models have a set of parameters that represent the physical properties of the systems, such as kinetic constants and reaction rates. In general, the development of these models requires two steps: model structure construction and parameter estimation. The models are often constructed with time derivative expressions, such as ordinary differential equations (ODEs), to describe the change of certain quantities of interest over time [1,2]. The model parameters are then estimated by simulating the actual processes obtained from experimental analyses [3–5]. However, because the differential equation model has many uncertain parameters and limited measurement data, parameter estimation is a major bottleneck in the development of useful biochemical models [6,7].

Optimization algorithms cannot deal with the high dimensionality of search space due to calculation complexity. One way to circumvent this difficulty is to simplify complicated systems biology models using model order reduction methods. Model order reduction methods reduce the number of states and parameters of dynamical systems that are defined by ODEs [8]. Lumping is one model order reduction method in which the original states of the model are lumped or merged to a reduced number of pseudo-states, resulting in a fewer equations and parameters but with effectively the same or similar input-output behavior. Proper lumping is a special case of lumping where each of the original states contributes to only one of the pseudostates of the reduced system thereby forming groups that retain a clear physical interpretation. With these methods, the reduced systems include less information, but are supposed to retain the basic features or properties of the original models. Although computational expense is saved, it is highly likely that the simplification loses critical information, especially if there is excessive simplification. Another strategy is to use divide-and-conquer methods, which decompose a large network of interest into smaller sub-networks [9,10]. For example, Voit and Almeida [11] developed an approach to transforming the problem into several sets of decoupled algebraic equations, being processed efficiently in parallel or sequentially, in large genetic network models. Kimura et al. [12] employed a cooperative co-evolutionary algorithm with a decomposition strategy to handle large S-system models with noisy time-series data. When there are no closed loops, Koh et al. [13] decomposed the network into small, independent sub-networks and estimated the parameters for each sub-network separately under the assumption that signals or mass flow in one direction. van Riel and Sontag [14] proposed a different approach to utilizing the modular structure of biochemical networks, providing the time courses of the intra-modular components that interact with neighboring modules. Those divide-and-conquer strategies, however, are not suitable for complex networks consisting of multiple closed or feedback loops, because dividing closed loops can change their intrinsic regulatory structures, greatly altering their dynamic features and the sensitivity of search parameters. Recently, to handle this difficulty, Maeda et al. [5] employed flux module decomposition that separates a complex, large-scale dynamic model into multiple flux modules without destroying its basic control architectures. However, it assumes that all parameters are necessary without accounting for differences in uncertainty of parameters.

To circumvent the aforementioned issues, we propose a divide-and-conquer approach to avoiding unnecessary information loss while estimating high-dimensional parameters efficiently. To do this, we first divide a large complete system into sub-systems so that each subsystem has a smaller, manageable number of differential equations. Then we estimate parameters for each sub-system, followed by refinement of the estimates through communication among subsystems. The success of the proposed algorithm depends on how the complete system is divided into small sub-systems.

We illustrate our proposed approaches with a simple three-compartment model. Its system of ordinary differential equations (ODEs) is as follows:

$$\frac{dx(t)}{dt}=-Ka\cdot x(t);$$

$$\frac{dy(t)}{dt}=Ka\cdot x(t)-Kb\cdot y(t)+Kb\cdot z(t);$$

$$\frac{dz(t)}{dt}=Kb\cdot y(t)-Kb\cdot z(t)-Kc\cdot z(t)$$

Where (Ka,Kb,Kc) are the parameters to estimate (i.e., Ka,Kb,Kc are the absorption rate, the distribution rate, the elimination rate constants, respectively); and (*x*(*t*), *y*(*t*), *z*(*t*))_*t* = 0_ = (0, 0, 0). Its graphical representation is shown in Figure 1a. Using this model, we investigated the performance of the proposed approach in a simulation study. We generated 100 simulations and estimated the parameters using 1) a conventional approach (ONE) and 2) a divide-and-conquer approach (DAQ). The brief schematic representation of DAQ can be seen in Figure 1b.

As for DAQ, the parameters (Ka, Kb) are first estimated given Kc and then the parameters (Kb, Kc) are estimated given Ka. This procedure was repeated until convergence. Table 1 displays the results of 100 simulation studies with mean squared errors (MSEs) and estimates’ bias by three levels of measurement errors. The performance of DAQ is comparable to that of ONE, and, in some cases, the biases of DAQ are smaller than these of ONE in Table 1.

It is worth noting that, as the whole model is divided into smaller models, the computation expense decreases, but the information loss increases. For this reason, it is important to ensure that the decomposition is optimal, and future work will further need to find out the relationship between the decomposition and the information loss. Overall, as shown in the limited simulation study, the proposed approach preserves important properties of the original model and thereby increases the quality of the biochemical networks due to the property that the proposed approach does not depend on simplification. Furthermore, the proposed parameter estimation approach can be easily applied to other high-dimensional data such as genomics, transcriptomics, proteomics, and metabolomics. Therefore, the proposed work will benefit for many types of high-dimensional studies.

## Figures and Tables

**Figure 1:**
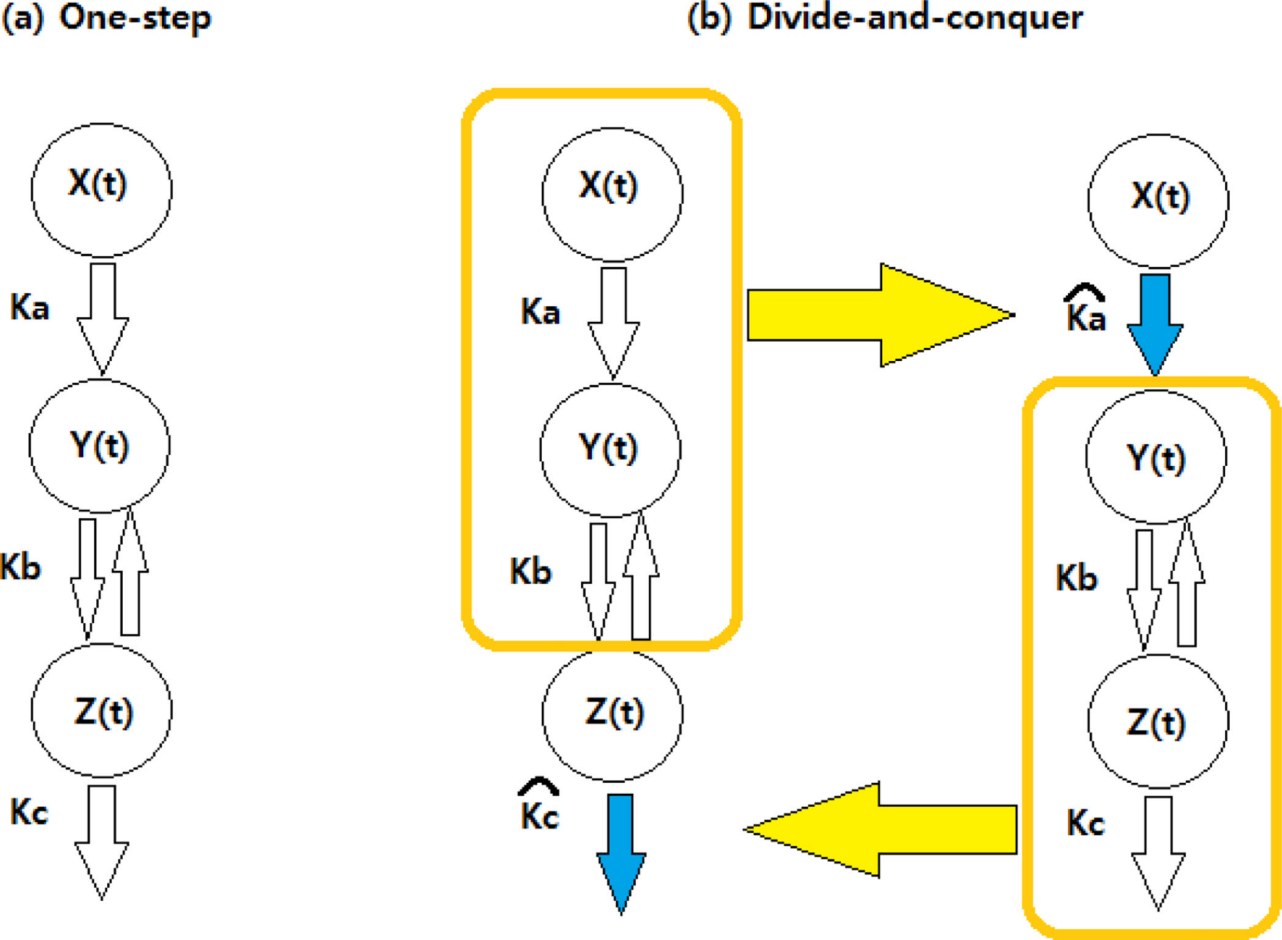
Graphical representation of three compartmental models.

**Table 1:** Results of 100 simulations of ONE and DAQ.

		σ=0.1		σ=0.5		σ=1.0	
	TRUE	DAQ	ONE	DAQ	ONE	DAQ	ONE
		Mean	Mean	Mean	Mean	Mean	Mean
		(SD)	(SD)	(SD)	(SD)	(SD)	(SD)
MSE		0.009	0.009	0.216	0.212	0.875	0.859
		−0.004	−0.004	−0.098	−0.096	−0.365	−0.358
		Bias	Bias	Bias	Bias	Bias	Bias
		(SD)	(SD)	(SD)	(SD)	(SD)	(SD)
Ka	log(0.8)	0.003	0.001	0.025	0.012	0.048	0.022
		−0.006	−0.007	−0.043	−0.045	−0.084	−0.09
Kb	log(10)	0.005	0.001	0.045	0.034	0.104	0.073
		−0.011	−0.021	−0.073	−0.067	−0.134	−0.218
Kc	log(8)	−0.002	−0.001	−0.021	−0.026	−0.038	−0.047
		−0.006	−0.014	−0.044	−0.044	−0.086	−0.123
